# Assessment of synthetic post-therapeutic OCT images using the generative adversarial network in patients with macular edema secondary to retinal vein occlusion

**DOI:** 10.3389/fcell.2025.1609567

**Published:** 2025-06-04

**Authors:** Shi Feng, Jingyuan Yang, Xinyu Zhao, Jianchun Zhao, Yunfeng Du, Weihong Yu, Dayong Ding, Xirong Li, Youxin Chen

**Affiliations:** ^1^ Ophthalmology Department, Peking Union Medical College Hospital, Beijing, China; ^2^ Vistel AI Lab, Visionary Intelligence Ltd, Beijing, China; ^3^ Key Lab of DEKE, Renmin University of China, Beijing, China

**Keywords:** generative adversarial networks, retinal vein occlusion, anti-vascular endothelial growth factor, optical coherent tomography, therapeutic efficacy prediction

## Abstract

**Aims:**

The aim of this study is to generate post-therapeutic optical coherence tomography (OCT) images based on pre-therapeutic OCT by using generative adversarial networks (GANs). The synthetic images enable us to predict the short-term therapeutic efficacy of intravitreal injection of anti-vascular endothelial growth factor (VEGF) in retinal vein occlusion (RVO) patients.

**Methods:**

The study involved patients with RVO who received intravitreal anti-VEGF injection from 1 November 2018 to 30 November 2019. The OCT images taken before and shortly after treatment, with an interval of 4–8 weeks, were collected and randomly divided into the training set and test set at a ratio of approximately 3:1. The model is constructed based on the pix2pixHD algorithm, and synthetic OCT images are evaluated in terms of the picture quality, authenticity, the central retinal thickness (CRT), the maximal retinal thickness, the area of intraretinal cystoid fluid (IRC), and the area of subretinal fluid (SRF). Three supporting models, namely, the macular detection model, retinal stratification model, and lesion detection model, were constructed. Segmentation of macular location, retinal structure, and typical lesions were added to the input information. After verifying their accuracy, supporting models were used to detect the CRT, the maximal retinal thickness, IRC area, and SRF area of synthetic OCT images. The output predictive values are compared with real data according to the annotation on the real post-therapeutic OCT images.

**Results:**

A total of 1,140 pairs of pre- and post-therapeutic OCT images obtained from 95 RVO eyes were included in the study, and 374 images were annotated. Of the synthetic images, 88% were considered to be qualified. The accuracy of discrimination of real versus synthetic OCT images was 0.56 and 0.44 for two retinal specialists, respectively. The accuracy to predict the treatment efficacy of CRT, the maximal retinal thickness, IRC area, and SRF area was 0.70, 0.70, 0.92, and 0.78, respectively.

**Conclusion:**

Our study proves that the GAN is a reliable tool to predict the therapeutic efficacy of anti-VEGF injections in RVO patients. Evaluations conducted both qualitatively and quantitatively indicated that our model can generate high-quality post-therapeutic OCT images. Consequently, it has great potential in predicting the treatment efficacy and providing guidance to clinical decision-making.

## 1 Introduction

Retinal vein occlusion (RVO) is a significant cause of vision loss in elderly individuals worldwide and is the second-most common cause of vision loss due to retinal vascular disorders (Rogers et al., 2010; Romano et al., 2023). Central retinal vein occlusion (CRVO) is caused by blockage of the central retinal vein, usually due to thrombus formation; whereas branch retinal vein occlusion (BRVO) is caused by blockage of one of the branches of the central retinal vein, often occurring at arteriovenous crossings. Macular edema (ME) is a common complication of RVO, which severely affects central vision. ME is manifested as central retinal thickness (CRT) and the presence of intraretinal cystoid fluid (IRC) and subretinal fluid (SRF). The primary goal in the treatment of patients with RVO is to maintain central vision, with a particular focus on minimizing or preventing the formation of macular edema (Schmidt-Erfurth et al., 2019; Hogg et al., 2021). Current evidence suggests that intravitreal injection of anti-vascular endothelial growth factor (VEGF) is most efficient against ME-related visual impairment (Zhang, Liu, and Sang, 2022). Optical coherence tomography (OCT) images provide high-resolution image modality for quantifying retinal thickening and fluid accumulation and monitoring the treatment efficacy (Rayess et al., 2019). However, the response to anti-VEGF treatment shows significant heterogeneity in clinical practice (Tao et al., 2024; Wecker et al., 2017; Korobelnik et al., 2021), making it difficult to predict anatomic changes after anti-VEGF treatment.

Generative adversarial networks (GANs) involve a zero-sum competition between a generative model, which generates images, and a discriminative model, which evaluates whether the image came from real training data rather than being generated (Waisberg et al., 2025). It has been harnessed for image-to-image translation in ophthalmology to predict the responses to treatment by generating individualized post-therapeutic OCT images after anti-VEGF treatment for fundus diseases such as age-related macular degeneration (AMD) (Liu et al., 2020), RVO (Xu et al., 2022), and diabetic macular edema (DME) (Liu et al., 2023; Baek et al., 2024). Yet previous studies did not develop quantitative methods to assess the quality of synthetic images in an anatomical perspective. Herein, we established a series of supporting deep learning models to further evaluate the predictive performance of the GAN model so as to certify that our model can generate high-quality post-therapeutic OCT images.

## 2 Materials and methods

### 2.1 Study design and participants

Patients with RVO complicated by ME who underwent intravitreal anti-VEGF drug therapy at the Department of Ophthalmology, Peking Union Medical College Hospital, from November 2018 to November 2019, were retrospectively included in the study. The inclusion criteria were as follows: (1) patients diagnosed with ME secondary to RVO. The diagnosis was independently confirmed by at least two retinal specialists. (2) Patients who were administered intravitreal injections of anti-VEGF drugs. (3) Pre-therapeutic and post-therapeutic retinal OCT images were obtained within 4–8 weeks. The exclusion criteria were as follows: (1) a history of previous intraocular operation, laser photocoagulation, or intraocular injections of medications other than anti-VEGF agents. (2) A history of other ocular disorders, including glaucoma, pathological myopia, age-related macular degeneration, and other disorders involving systemic diseases.

This study was approved by the Clinical Research Ethical Committee of Peking Union Medical College Hospital, Chinese Academy of Medical Sciences (Project Number: S-K631). The research implementation adhered to the principles of the Declaration of Helsinki.

### 2.2 Dataset creation

#### 2.2.1 Pairs of OCT images

Pre-therapeutic and post-therapeutic swept-source OCT (SS-OCT) images were captured in a 16-line 9-mm radial macula pattern by a Topcon Deep Range Imaging (DRI) OCT Triton device (Topcon, Tokyo, Japan) with a resolution of either 1,024 × 875 pixels or 1,024 × 992 pixels. The images before and after the treatment were scanned at the same location using the follow-up mode and were matched by two retinal specialists. Images with motion artifacts or insufficient quality for clinical assessment were excluded. The OCT image pairs were randomly split into the training set and test set at a ratio of approximately 3:1. Double checks were made to ensure that images from the same patient were not distributed to the training set and test set simultaneously. All OCT images included in the study were anonymized to protect patient privacy.

#### 2.2.2 Annotation of OCT images

The OCT images were subjected to a secondary screening to identify those with the following characteristics for further image annotation: (1) OCT images with typical lesions of IRC and SRF. (2) OCT images with the maximum CRT measurement from the 16-line pre-therapeutic scans, along with its corresponding post-therapeutic image. Taking sample balance and training efficiency into consideration, no more than four pairs of OCT images were selected for each eye.

The following annotations were obtained to provide additional segmentation information: (1) normal retinal structures: upper limit of the inner limiting membrane (ILM), upper limit of the retinal pigment epithelium (RPE), and lower limit of the choroid. (2) Retinal thickness-related structures: central foveal position. (3) Major lesions: IRF and SRF.

The central foveal position was marked using a circular point, while the normal retinal structures, IRF and SRF, were annotated at the pixel level using multi-point polygon annotation. High-quality segmentation was accomplished by two retinal specialists, and the findings were cross-verified. Disagreements between specialists were resolved through consultation with a senior retinal specialist. Annotated OCT images were separated randomly into the training set and test sets at a ratio of 3:1. Three supporting models were developed based on annotated OCT images. No images of the same patient were simultaneously assigned to the two data sets.

### 2.3 Deep learning framework

All experiments were conducted with PyTorch deep learning framework (version 1.1.0) and Python (version 3.5) using the Linux operating system, Intel® Xeon® CPU E5-2680 v3 @2.50 GHz, and GeForce RTX 2080 Ti.

#### 2.3.1 Image synthesis model training

**FIGURE 1:**
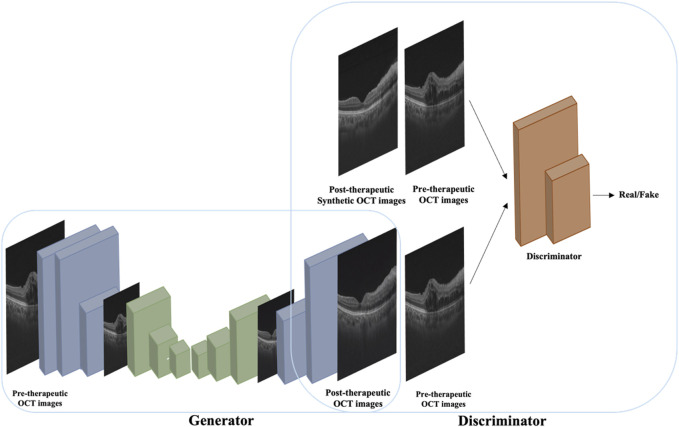
A conceptual illustration of the pix2pixHD-based solution used in this study for generating post-therapeutic OCT images from pre-therapeutic OCT images.

The image synthesis model was constructed based on pix2pixHD, a variant of conditional GAN, which consists of a coarse-to-fine generator and multiscale discriminators to generate high-resolution images. The network framework of the pix2pixHD model is shown in Figure 1. Using pairs of real pre-therapeutic and post-therapeutic images as input images, the image synthesis model was trained to generate a synthetic post-therapeutic OCT image as output information. The discriminative model was employed to distinguish the real post-therapeutic OCT images from the synthetic fake post-therapeutic OCT images. After the termination of the training, the generator network is capable of translating any given pre-therapeutic OCT image into the synthetic post-therapeutic image.

#### 2.3.2 Supporting model training and evaluation

Four anatomical parameters, namely, the central retinal thickness (CRT), the maximal retinal thickness, the area of IRC, and the area of SRF, were measured in order to comprehensively evaluate the synthetic images. Three supporting frameworks, the macular detection model, retinal stratification model, and lesion detection model, were constructed correspondingly.

##### 2.3.2.1 Macular detection model training and evaluation

The high-resolution networks (HRNets) algorithm yielded satisfactory performance in preservation of accurate position and was, therefore, adopted to detect the position of macula. The model took OCT image pairs and abscissa of macular annotations from the training set as input information and output the accurate horizontal ordinate of the macular position.

The difference between the gold-standard label and the predicted values was calculated as the data sample, and a 95% confidence interval was computed. The accuracy of the model is calculated by the percentage of correctly identified macular positions.

##### 2.3.2.2 Retinal stratification model training and evaluation

The U-Net algorithm gained wide application in the field of image segmentation and achieved better results even with relatively sparse annotation data due to the combination of the encoder and decoder. The retinal stratification model was constructed using U-Net and was trained based on OCT image pairs and segmentation information of ILM, RPE, and choroid annotated by retinal specialists. It ultimately outputs three categories, which were named class 0 (background), class 1 (from the upper limit of ILM to the upper limit of RPE), and class 2 (from the upper limit of RPE to the lower limit of choroid). The retinal stratification model has laid the foundation for precise measurement of CRT and maximal retinal thickness.

The performance of the test set in the trained retinal stratification model was compared with the results of two retinal specialists in terms of recall, precision, intersection over union (IOU), and Dice coefficient.

##### 2.3.2.3 Lesion detection model training and evaluation

The U-Net algorithm was applied to develop the lesion detection model in order to achieve accurate measurement of IRC and SRF areas. OCT images from the training set and segmentation of IRC and SRF were inputted. Three categories, class 0 (background), class 1 (SRF), and class 2 (IRC), were outputted during the training process.

The constructed model recognized the OCT images of the test set and categorized each pixel point in the image into three classes. The lesions annotated by ophthalmologists were considered the ground truth labels. Subsequently, recall, precision, IOU, and Dice coefficient were calculated.

### 2.4 Evaluation of synthetic images

#### 2.4.1 Quality of synthetic images

Synthetic images were assessed by two retinal specialists independently to determine whether they are qualified for clinical interpretation, such as retinal structure defects and repeated retinal structure. Notably, synthetic images with insufficient quality were excluded for further evaluation.

#### 2.4.2 Authenticity of synthetic images

OCT images that adhered to the basic image regulation underwent evaluation of authenticity. All images were processed to remove irrelevant information, including contrast differences and pixel variations. The real post-therapeutic images and corresponding synthetic post-therapeutic images were simultaneously displayed to two retinal specialists in a random sequence without any mark, while the pre-therapeutic images were labeled for reference. The two retinal specialists were required to identify the synthetic images. The proportion of correct judgments made by the two doctors was then calculated separately.

#### 2.4.3 Structural evaluations of synthetic images

The verified macular detection model, retinal stratification model, and lesion detection model were applied to recognize post-therapeutic OCT images generated from the GAN model, and predicted values of CRT, maximum retinal thickness, SRF area, and IRC area were obtained. The retinal thickness and area annotated by retinal specialists on real post-treatment images were regarded as the gold standard.

Quantitative evaluation of synthetic images was conducted by comparing the predicted values with the gold standard. If the predicted value of the synthetic post-therapeutic OCT image exceeded the gold standard value of the pre-therapeutic OCT image by 10%, it was recognized as an “increase.” If the gold standard value represented a 10% increase over the predicted value, it was regarded as a “decrease.” If the predictive value and gold standard measurements differed by ≤10% (absolute difference), two retinal specialists independently evaluated the OCT images and output data to determine the treatment trend. The absence of SRF and IRC both before and after the treatment was defined as “no change” (both 0). The gold standard value of the real post-therapeutic OCT image was also compared with that of the pre-therapeutic OCT image. The accuracy to predict the treatment efficacy was referred to as the proportion of synthetic post-therapeutic images that showed the same trend as the real post-therapeutic images, compared with the pre-therapeutic images.

### 2.5 Evaluation metrics and statistical analysis

Open-source Python library scipy.stats (version 0.14.0, The Scipy community) was applied for statistical analysis. For categorical variables, classification accuracy and the 95% confidence interval (CI) were calculated. The Shapiro test was used to determine whether the differences between the gold standard and the predicted values followed a normal distribution. A paired t-test was employed for significance analysis if the variables were identified as normally distributed. Otherwise, a Wilcoxon test was used for significance analysis. A p-value less than 0.05 was considered of significant differences.

## 3 Result

### 3.1 Dataset of synthetic images

According to the inclusion and exclusion criteria, the study finally included 726 pairs of OCT images for BRVO eyes and 307 pairs for CRVO eyes. A total of 570 pairs of OCT images from 49 cases of BRVO eyes and 226 pairs of OCT images from 21 cases of CRVO eyes were included in the training set. Additionally, 157 pairs of OCT images from 15 cases of BRVO eyes and 80 pairs of OCT images from eight cases of CRVO eyes were assigned into the test set.

### 3.2 Quality of synthetic images

**FIGURE 2:**
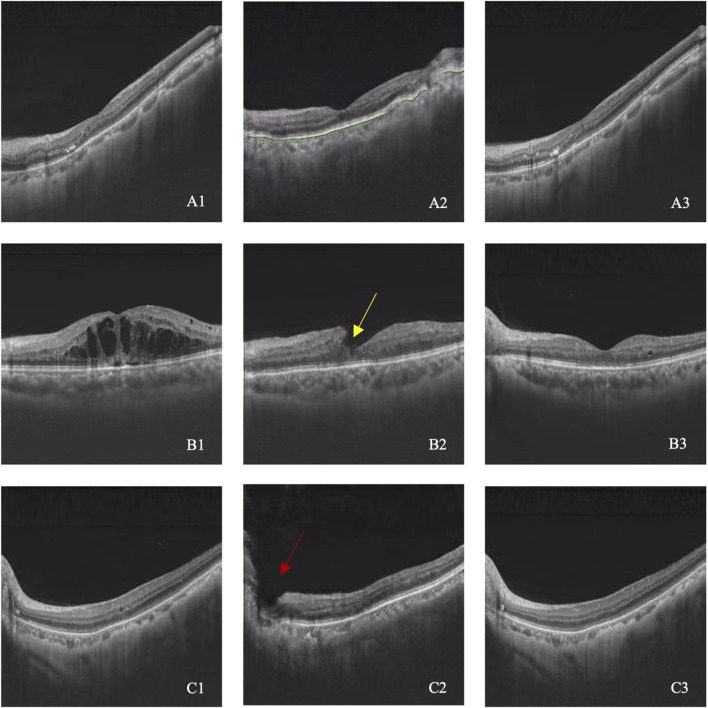
**(A–C)** illustrated three cases with image quality issues. A1–C1 represent pre-therapeutic OCT images, A2–C2 represent synthetic post-therapeutic OCT images, and A3–C3 represent the real post-therapeutic OCT images. RPE discontinuity is shown in A2 (green line), retinal neuroepithelium discontinuity is shown in B2 (yellow arrow), and the entire retinal discontinuity is shown in C2 (red arrow).

Among 237 generated post-therapeutic OCT images, 29 images from 10 cases failed to meet the basic image regulation and were excluded from further evaluation. Therefore, 208 out of 237 images (87.86%) were identified as qualified for clinical interpretation. All 29 unqualified images were related to abrupt structural rupture, with 16 images showing retinal neuroepithelium discontinuity, 8 images showing retinal pigment epithelium discontinuity, and 5 images showing entire retinal discontinuity. Three cases of incompetent synthetic images are shown in Figure 2.

### 3.3 Authenticity of synthetic images

**FIGURE 3:**
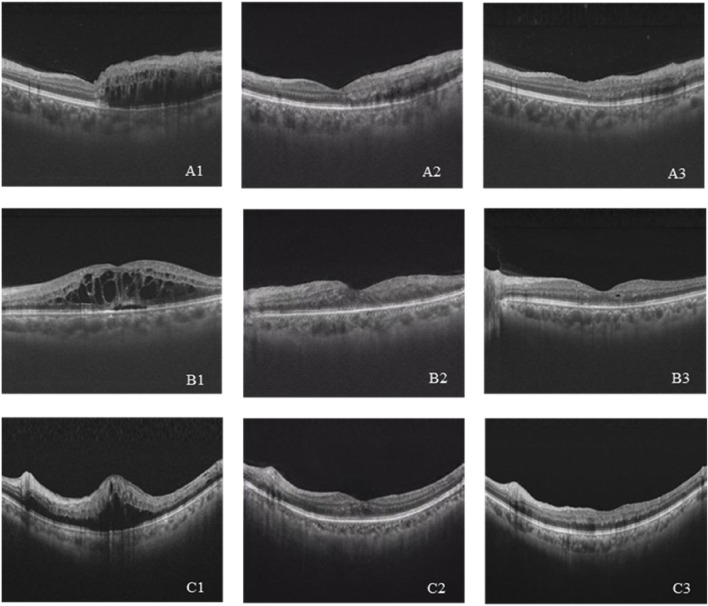
**(A–C)** Three cases of pre-therapeutic, real post-therapeutic, and synthetic post-therapeutic OCT images. A1–C1 represent pre-therapeutic OCT images, A2–C2 represent synthetic post-therapeutic OCT images, and A3–C3 represent real post-therapeutic OCT images.

A total of 208 synthetic images and real images were shown to the retinal specialists to validate the authenticity. The rate to discriminate between synthetic and real images successfully was 0.56 (95% CI 0.47–0.65) for specialist 1 and 0.44 (95% CI 0.34–0.54) for specialist 2. It is challenging to distinguish between synthetic and real images, indicating that the GAN model has achieved eligible results. Six cases of pre-therapeutic, real post-therapeutic, and synthetic post-therapeutic OCT images are displayed in Figure 3.

### 3.4 Verification of supporting models

#### 3.4.1 Dataset pf supporting models

After a second screening, 280 OCT images from 64 eyes with BRVO and 94 OCT images from 29 eyes with CRVO were included in the annotation dataset. Less than four pairs of OCT images were selected for each eye. Two retinal disease specialists were assigned to annotate the OCT images, and there were no disagreements upon cross-validation. A total of 226 OCT images from 49 eyes with BRVO and 74 OCT images from 21 eyes with CRVO were involved in the training set. The test set consisted of 54 OCT images from 15 eyes with BRVO and 20 OCT images from 8 eyes with CRVO.

#### 3.4.2 Verification of the macular detection model

**FIGURE 4:**
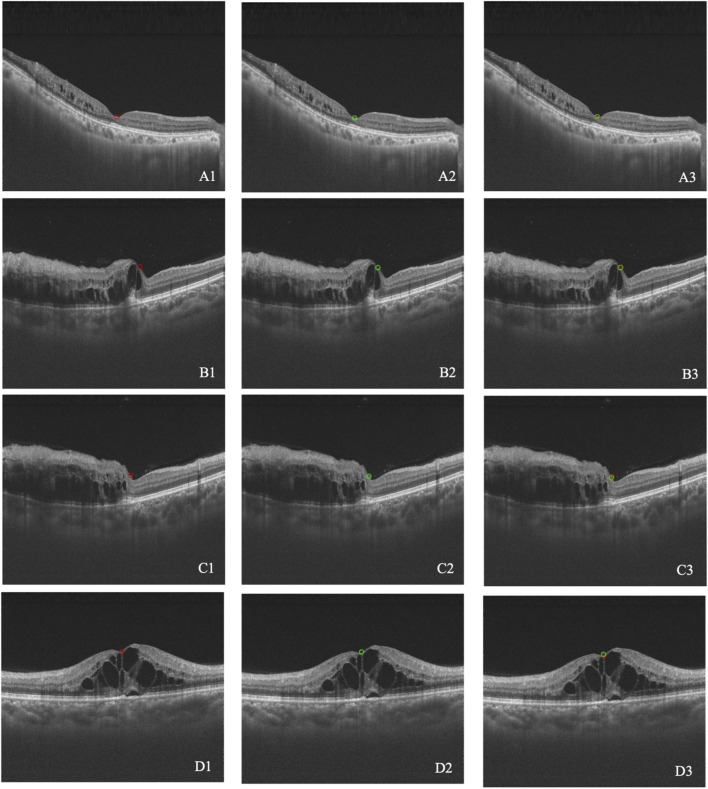
**(A–D)** Four cases of macular detection. A1–D1 represent the macular position annotated by retinal specialists (red circle). A2–D2 represent the macular position detected by the model (green circle). A3–D3 show the overlap between the annotated and detected positions.

The difference of the macula position between the synthetic image and the real image was calculated. The mean difference was 0.0204 ± 0.0604 for BRVO eyes, 0.0060 ± 0.0054 for CRVO eyes, and 0.0165 ± 0.0521 for RVO eyes. The accuracies of the macular detection model were 75.93% for BRVO eyes, 80.00% for CRVO eyes, and 77.03% for all RVO eyes. Overall, the model demonstrated relatively accurate detection of the macular center position. Figure 4 illustrates four cases of the detection.

#### 3.4.3 Verification of the retinal stratification model

**FIGURE 5:**
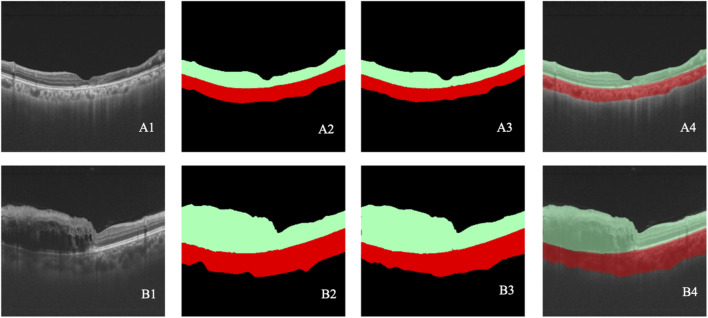
**(A–B)** Two cases of retinal stratification. A1–B1 represent the original OCT images. A2–B2 represent the retinal stratification annotated by retinal specialists. A3–B3 represent retinal stratification detected by the model. A4–B4 show the overlap between the annotated and detected stratification. Black indicates class 0, green indicates class 1, and red indicates class 2.

Table 1 reports the performance of the retinal stratification model, and Figure 5 illustrates two examples of the stratification. Among them, class 1 represented the thickness of the retina, which was of great concern in our study. The recall, accuracy, IOU, and Dice coefficient of class 1 were 0.99, 0.99, 0.98, and 0.99, respectively, for BRVO, CRVO, and RVO eyes. Therefore, the retinal stratification model demonstrated accurate identification for retinal thickness and was eligible.

**TABLE 1:** Performance of the retinal stratification model.

Diagnosis	Categories	Recall	Accuracy	IOU	Dice coefficient
BRVO	Class 0	0.9967	0.9893	0.9861	0.9930
	Class 1	0.9872	0.9899	0.9773	0.9885
	Class 2	0.9258	0.9737	0.9032	0.9491
	Average	0.9699	0.9843	0.9555	0.9769
CRVO	Class 0	0.9836	0.9959	0.9797	0.9897
	Class 1	0.9890	0.9891	0.9783	0.9891
	Class 2	0.9695	0.8917	0.8674	0.9290
	Average	0.9807	0.9589	0.9418	0.9693
RVO	Class 0	0.9932	0.9910	0.9843	0.9921
	Class 1	0.9877	0.9896	0.9776	0.9887
	Class 2	0.9374	0.9497	0.8930	0.9435
	Average	0.9728	0.9768	0.9517	0.9748

#### 3.4.4 Verification of the lesion detection model

**FIGURE 6:**
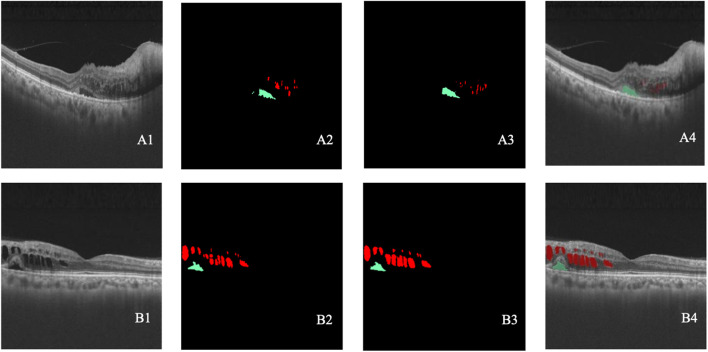
**(A–B)** Two cases of lesion detection. A1–B1 represent the original OCT images. A2–B2 represent the lesions annotated by retinal specialists. A3–B3 represent the lesion detected by the model. A4–B4 show the overlap between the annotated and detected lesions. Black indicates class 0, green indicates class 1, and red indicates class 2.

In the lesion detection model, class 1 (SRF) and 2 (IRC) represented the quantitative areas of lesions, which was highly indicative of treatment efficacy. As for eyes with BRVO, the recall, accuracy, IOU, and Dice coefficient were 0.87, 0.84, 0.76, and 0.85 for class 1 and 0.90, 0.66, 0.61, and 0.76 for class 2, respectively. When it came to eyes with CRVO, the recall, accuracy, IOU, and Dice coefficient were 0.68, 0.87, 0.62, and 0.76 for class 1 and 0.63, 0.94, 0.61, and 0.75 for class 2, respectively. Overall, the lesion detection model exhibited competent recognition capabilities for IRC and SRF lesions, meeting the requirements of the study. Table 2 presents the results of the lesion detection model, and Figure 6 displays two examples of the detection.

**TABLE 2:** Performance of the lesion detection model.

Diagnosis	Categories	Recall	Accuracy	IOU	Dice coefficient
BRVO	Class 0	0.9979	0.9995	0.9973	0.9987
	Class 1	0.8743	0.8360	0.7463	0.8548
	Class 2	0.8973	0.6570	0.6111	0.7586
	Average	0.9232	0.8308	0.7849	0.8707
CRVO	Class 0	0.9994	0.9950	0.9944	0.9972
	Class 1	0.6786	0.8743	0.6183	0.7641
	Class 2	0.6308	0.9373	0.6052	0.7541
	Average	0.7696	0.9355	0.7393	0.8385
RVO	Class 0	0.9983	0.9983	0.9965	0.9983
	Class 1	0.8394	0.8413	0.7247	0.8404
	Class 2	0.7554	0.7578	0.6085	0.7566
	Average	0.8643	0.8658	0.7766	0.8651

### 3.5 Structural evaluations of synthetic images

#### 3.5.1 Dataset of structural evaluation

The annotations made by two retinal specialists on real post-therapeutic OCT images were considered the gold standard. Therefore, only images with annotations could be used for structural evaluations. Since the evaluation required the application of three supporting models, the images from the training sets of the aforementioned models were not included in the test set to ensure the accuracy of evaluations. Therefore, the division of the structural evaluations dataset should be consistent with the above models. A total of 37 post-therapeutic OCT images generated by the GAN model were input into the macular detection model, retinal stratification model, and lesion detection model. The following parameters were outputted: (1) CRT: the macular detection model located the macular position, and the retinal stratification model identified the corresponding retinal thickness. (2) Maximal retinal thickness: the retinal stratification model recognized retinal thickness, and maximal thickness within a single OCT image was reported. (3) SRF area: the lesion detection model identified SRF lesions and outputted the specific area of the SRF. (4) IRC area: the lesion detection model identified IRC lesions and outputted the specific area of the IRC.

#### 3.5.2 Evaluation of CRT

Evaluation of the predictive performance on the trend of CRT is shown in Table 3. The accuracy in predicting the trend of CRT changes in post-therapeutic OCT images was 0.70. There were no significant differences between the predicted values and the gold standard CRT for BRVO, CRVO, and RVO eyes, as shown in Table 9. The distribution of CRT is shown in Supplementary Figure S2. Bland–Altman analysis demonstrated that 89.2% predicted values were distributed within the 95% limits of agreement (LoA). This indicated that our model could predict changes in CRT after treatment with anti-VEGF agents with precision.

**TABLE 3:** Evaluation of the predictive performance on the trend of CRT.

		Comparison of real post-therapeutic images with pre-therapeutic images		
		Decrease in CRT	Increase in CRT	Total number
Comparison of synthetic post-therapeutic images with pre-therapeutic images	Decrease of CRT	23	4	27
	Increase of CRT	7	3	10
	Total number	30	7	37

#### 3.5.3 Evaluation of maximal retinal thickness

Evaluation of the predictive performance on the trend of maximal retinal thickness is shown in Table 4. The accuracy in predicting the trend of maximal retinal thickness changes in post-therapeutic OCT images was 0.70. There were no significant differences between the predicted values and the gold standard maximal retinal thickness for BRVO, CRVO, and RVO eyes, as shown in Table 9. The distribution of maximal retinal thickness is shown in Supplementary Figure S2. Bland–Altman analysis demonstrated that 94.6% predicted values were distributed within the 95% LoA. The predictive performance of our model on changes in maximal retinal thickness was qualified.

**TABLE 4:** Evaluation of predictive performance on the trend of maximal retinal thickness.

		Comparison of real post-therapeutic images with pre-therapeutic images		
		Decrease in maximal retinal thickness	Increase in maximal retinal thickness	Total number
Comparison of synthetic post-therapeutic images with pre-therapeutic images	Decrease in maximal retinal thickness	24	6	30
	Increase in maximal retinal thickness	5	2	7
	Total number	28	8	37

#### 3.5.4 Evaluation of the SRF area

The accuracy in predicting the presence of SRF in post-therapeutic OCT images was 0.86, as shown in Table 5. The specificity was 0.97 (95% CI 0.82–1), while the sensitivity was 0 (95% CI 0–0.60). Out of the 37 synthetic OCT images, only one revealed SRF. This suggested that our model had limited capability in predicting SRF after treatment, highly likely due to the limited dataset of SRF. Evaluation of the predictive performance on the trend of SRFarea is shown in Table 6. The accuracy in predicting the trend in SRF area changes in post-therapeutic OCT images was 0.92. It yielded satisfactory achievements in predicting a decrease or no change in SRF but made errors in predicting an increase in three cases. There were no significant differences between the predicted values and the gold standard for the SRF area in BRVO, CRVO, and RVO eyes, as shown in Table 9. The distribution of the SRF area is shown in Supplementary Figure S2. Bland–Altman analysis demonstrated that 97.3% predicted values were distributed within the 95% LoA.

**TABLE 5:** Evaluation of predictive performance on the presence of SRF.

		Real post-therapeutic images		
		Without SRF	With SRF	Total number
Synthetic post-therapeutic images	Without SRF	32	4	36
	With SRF	1	0	1
	Total number	33	4	37

**TABLE 6:** Evaluation of the predictive performance on the trend of the SRF area.

		Decrease in SRF area	Comparison of real post-therapeutic images with pre-therapeutic images		
			No change	Increase in SRF area	Total number
Comparison of synthetic post-therapeutic images with pre-therapeutic images	Decrease in SRF area	10	0	0	10
	No change	0	24	3	27
	Increase in SRF area	0	0	0	0
	Total number	10	24	3	37

#### 3.5.5 Evaluation of the IRC area

The accuracy in predicting the presence of IRC in post-therapeutic OCT images was 0.65, as shown in Table 7. The specificity was 0.86 (95% CI 0.64–0.96), but the sensitivity was 0.33 (95% CI 0.13–0.61), which was slightly superior compared with that for the SRF area. Evaluation of the predictive performance on the trend of IRCarea is shown in Table 8. The accuracy in predicting the trend of IRC area changes in post-therapeutic OCT images was 0.78. Among 37 cases; our model mistook an increase for a decrease or no change in six cases. The GAN model tended to underestimate the IRC area in post-therapeutic images. There were no significant differences between the predicted values and the gold standard for the IRC area in CRVO eyes, as depicted in Table 9. However, it failed to predict the IRC area accurately in BRVO and RVO eyes. The distribution of the IRC area is shown in Supplementary Figure S2. Bland–Altman analysis demonstrated that 94.6% predicted values were distributed within the 95% LoA.

**TABLE 7:** Evaluation of predictive performance on the presence of IRC.

		Real post-therapeutic images		
		Without IRC	With IRC	Total number
Synthetic post-therapeutic images	Without IRC	19	10	29
	With IRC	3	5	8
	Total number	22	15	37

**TABLE 8:** Evaluation of the predictive performance on the trend of IRC area.

		Decrease in IRC area	Comparison of real post-therapeutic images with pre-therapeutic images		
			No change	Increase in IRC area	Total number
Comparison of synthetic post-therapeutic images with pre-therapeutic images	Decrease in IRC area	23	1	5	29
	No change	0	5	1	6
	Increase in IRC area	1	0	1	2
	Total number	24	6	7	37

**TABLE 9:** Evaluation of the predictive performance on CRT, maximal retinal thickness, SRF area, and IRC area.

	Diagnosis	Average (standard deviation)		P-value (Shapiro test)	Significance test	P-value (significance)
CRT/μm	BRVO	Predicted values	75.48 (14.71)	0.0005	Wilcoxon	0.2028
		Gold standard	76.78 (41.39)			
	CRVO	Predicted values	87.80 (26.43)	0.0774	Paired t-test	0.8540
		Gold standard	91.50 (53.63)			
	RVO	Predicted values	78.81 (19.40)	0.0001	Wilcoxon	0.1697
		Gold standard	80.76 (45.50)			
Maximal retinal thickness/μm	BRVO	Predicted values	138.9 (26.14)	0.0555	Paired t-test	0.7345
		Gold standard	137.0 (33.81)			
	CRVO	Predicted values	156.9 (41.06)	0.0004	Wilcoxon	0.4922
		Gold standard	160.5 (55.19)			
	RVO	Predicted values	143.7 (31.92)	0.0007	Wilcoxon	0.2806
		Gold standard	143.4 (42.02)			
SRF area	BRVO	Predicted values	20.85 (106.3)	8.21e^−10^	Wilcoxon	0.2850
		Gold standard	100.5 (411.0)			
	CRVO	Predicted values	0 (0)	7.71e^−6^	Wilcoxon	0.1797
		Gold standard	99.45 (199.8)			
	RVO	Predicted values	15.22 (91.30)	2.81e^−11^	Wilcoxon	0.2249
		Gold standard	100.2 (366.2)			
IRC area	BRVO	Predicted values	51.63 (182.8)	9.12e^−7^	Wilcoxon	0.0018
		Gold standard	2780 (4,719)			
	CRVO	Predicted values	642.0 (1,590)	0.0001	Wilcoxon	0.3452
		Gold standard	8,391 (16,426)			
	RVO	Predicted values	211.2 (881.2)	1.40e^−9^	Wilcoxon	0.0014
		Gold standard	4,296 (9,767)			

## 4 Discussion

Considering the high cost of anti-VEGF medications and the burden of frequent follow-ups, individualized prediction of treatment efficacy is of great significance in clinical practice. Predicting the efficacy of anti-VEGF treatment in RVO patients facilitates the development of personalized treatment plans, benefiting patients, society, and healthcare providers.

However, how to evaluate the accuracy of synthetic images remains controversial. Previous research on the evaluation of AI-generated images mostly involved qualitative assessments, and retinal specialists were requested to distinguish between real and synthetic images (Zheng et al., 2020). Xu et al. developed a GAN-based prediction model to generate short-term post-therapeutic OCT images of RVO patients. In addition to authenticity evaluation, they conducted a structural evaluation experiment by measuring the CRT of the synthetic images and real images. There was no statistical difference in CMT between the synthetic and the real images (Xu et al., 2022). We made exploratory attempts to make elaborate lesion labels and construct three deep learning models in order to quantitatively assess the quality of the synthetic images on a structural perspective. The macular detection model and retinal stratification model showed remarkable achievements, while the lesion detection model exhibited slightly lower recall and accuracy on recognizing SRF and IRC. Nevertheless, our model still surpassed the performance of the previous research on quantifying the IRC and SRF in RVO patients (Schlegl et al., 2018). Although these supporting models showed promising results, the application of more models inevitably introduced more errors into the structural evaluation of the GAN model. In the structural evaluation, regarding the specific fluid area, there was no significant difference between the predicted values and the gold standard. However, in terms of the trend and the classification of fluid presence or absence, the sensitivity and specificity of several indicators were slightly lower. This might be related to the small size of the test set and the inherent biases in the samples.

This study innovatively utilized the GAN algorithm to predict the efficacy of anti-VEGF treatment in RVO patients. It was found that 88% of the synthetic images were of sufficient quality for further clinical interpretation. Retinal specialists reported difficulty in distinguishing between real and synthetic images. As for CRVO patients, there were no significant differences between the predicted values and the gold standard on CRT, retinal maximum thickness, SRF area, and IRC area. Similarly, for BRVO patients, there were no significant difference in the prediction of CRT, retinal maximum thickness, and SRF area. The accuracy of predicting the trend in CRT, retinal maximum thickness, SRF area, and IRC area after treatment was 0.70, 0.70, 0.92, and 0.78, respectively. With more clinical data and algorithm optimization, the GAN model holds the promise of bringing new insights into the dilemma of treatment selection for RVO patients.

This study has several limitations. First, the small sample that only included Chinese patients and the lack of an external validation dataset restrict the potential for generalization. Second, the functional evaluation of the predictive performance is still absent. Visual acuity is also what ophthalmologists and patients are concerned about. Further research could be conducted to cover more prognostic factors to make the model more applicable in predicting the visual acuity. Third, the research only evaluated short-term treatment outcomes after treatment with a single dose of anti-VEGF drugs. Though researchers realized the long-term prediction for patients with DME, it remains unclear whether long-term efficacy predictions for patients with RVO can be made. Previous studies have found that for CRVO patients, the optimal treatment effect is achieved at 12 months after initiation, while BRVO patients recover to optimal visual acuity after 24 months of treatment. Visual acuity and central retinal thickness then remain stable at the same level until the end of follow-up (Arrigo et al., 2021). Therefore, predicting the optimal treatment effect for patients before the initiation of anti-VEGF treatment would be more meaningful. Additionally, it should be noted that our study did not investigate several prognostic biomarkers associated with visual function, such as disorganization of retinal inner layers (DRIL) (Horozoglu et al., 2023). DRIL is correlated with areas of ischemic damage and loss of flow in the superficial, middle, and deep capillary plexuses, highlighting its potential as a biomarker for ischemic injury in RVO (Zhu et al., 2021; Munk et al., 2024). Previous studies have found the association of DRIL with poorer visual outcomes (Yang et al., 2024) and recurrence (Costa et al., 2021) in RVO-associated ME. Further annotations are required to involve those prognostic biomarkers, constructing a more comprehensive deep learning model.

## 5 Conclusion

Herein, our results prove that the GAN is a reliable tool to predict the therapeutic efficacy of anti-VEGF injections in RVO patients. Innovative quantitative evaluations were conducted with the assistance of supporting deep learning models, confirming the generation of high-quality post-therapeutic OCT images. Consequently, it has great potential in predicting treatment efficacy and providing guidance to clinical decision-making.

## Data Availability

The datasets presented in this article are not readily available because it contains private data. Requests to access the datasets should be directed to fengshi@pumch.cn.

## Supplementary material

SUPPLEMENTARY FIGURE S1 Workflow diagram of the synthetic OCT generation and evaluation.

SUPPLEMENTARY FIGURE S2 Structural evaluations of synthetic images. A1–D1 show the scatterplots of CRT, maximal retinal thickness, SRF area, and IRC area. A2–D2 show the Bland–Altman analysis of CRT (mean and SD of difference were 1.95 and 47.59, respectively), maximal retinal thickness (mean and SD of difference were −0.35 and 29.08, respectively), SRF area (mean and SD of difference were 85.00 and 386.66, respectively), and IRC area (mean and SD of difference were 4085.10 and 9909.70, respectively). The horizontal axis represents the average of the predicted values and gold standard. The vertical axis represents the difference between them. Notably, the predicted values and gold standard were not the absolute value of the thickness and area but the output data of the deep learning model without the unit.
