# Supplementary figures and images for: Assessment of synthetic post-therapeutic OCT images using the generative adversarial network in patients with macular edema secondary to retinal vein occlusion
Source: Front Cell Dev Biol. 2025 Jun 4;13:1609567. doi: 10.3389/fcell.2025.1609567 (PMC12174594; doi:10.3389/fcell.2025.1609567)

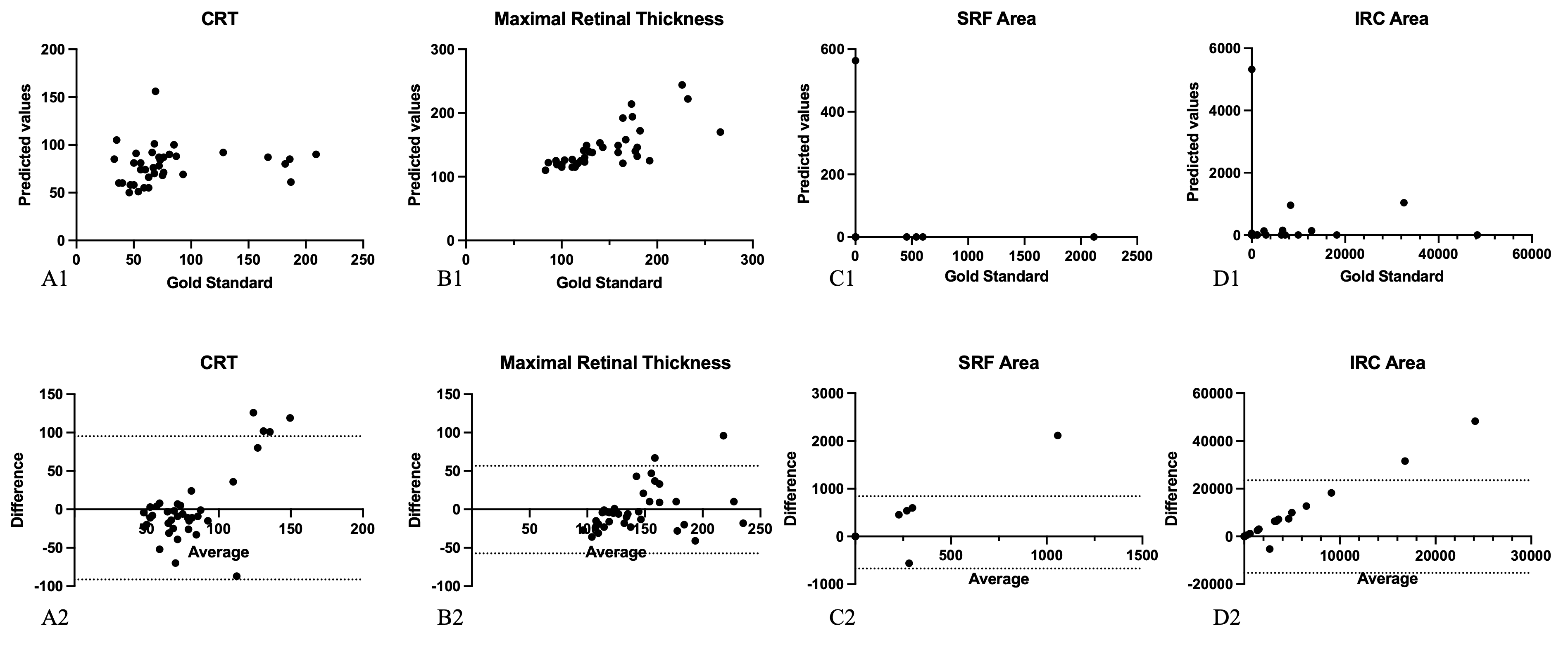

Supplement: Supplementary file 1 [file Image2.tif]

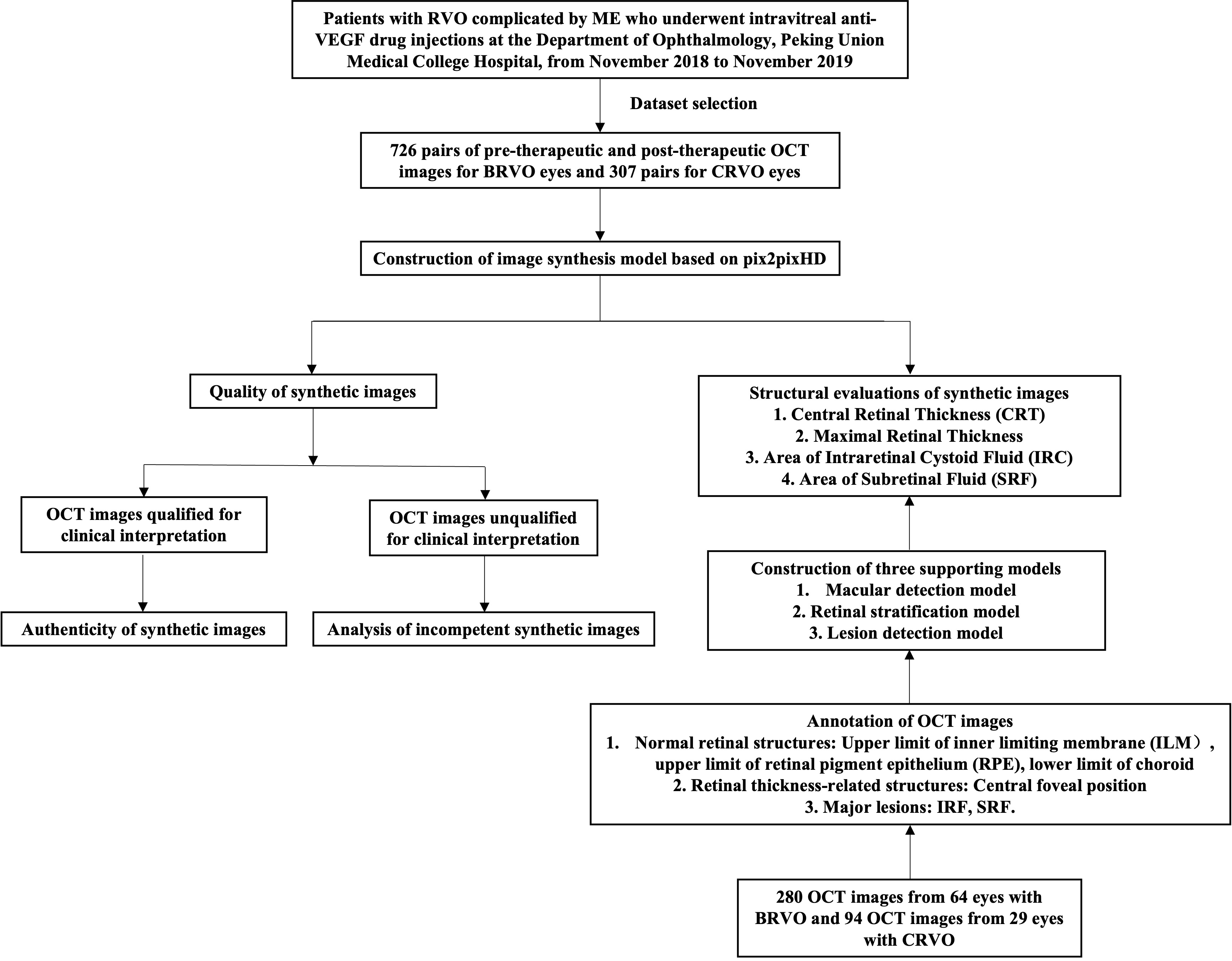

Supplement: Supplementary file 2 [file Image1.tif]
